# Cost-effectiveness of targeted thrombolytic therapy for stroke patients using multi-modal CT compared to usual practice

**DOI:** 10.1371/journal.pone.0206203

**Published:** 2018-10-23

**Authors:** Penny Reeves, Kim Edmunds, Christopher Levi, Longting Lin, Xin Cheng, Richard Aviv, Tim Kleinig, Kenneth Butcher, Jingfen Zhang, Mark Parsons, Andrew Bivard

**Affiliations:** 1 Health Research Economics, Hunter Medical Research Institute (HMRI), Newcastle, New South Wales, Australia; 2 School of Medicine and Public Health, University of Newcastle, Newcastle, New South Wales, Australia; 3 Department of Neurology, John Hunter Hospital, Newcastle, NSW, Australia; 4 Department of Neurology, Huashan Hospital, Fudan University, Shanghai, China; 5 Department of Medical Imaging, Sunnybrook Health Sciences Centre, and University of Toronto, Toronto, Canada; 6 Department of Neurology, Royal Adelaide Hospital, Adelaide, SA, Australia; 7 Division of Neurology, Department of Medicine, University of Alberta, Edmonton, Canada; 8 Department of Neurology, Baotou Central Hospital, Baotou, China; Harvard T.H. Chan School of Public Health, UNITED STATES

## Abstract

**Introduction:**

The use of multimodal computed tomography imaging (MMCT) in routine clinical assessment of stroke patients improves the identification of patients with large regions of salvageable brain tissue, lower risk for haemorrhagic transformation, or a large vessel occlusion requiring endovascular therapy.

**Aim:**

To evaluate the cost-effectiveness of using MMCT compared to usual practice for determining eligibility for reperfusion therapy with alteplase using real world data from the International Stroke Perfusion Imaging Registry (INSPIRE).

**Methods:**

We performed a cost-utility analysis. Mean costs and quality-adjusted life years (QALYs) per patient for two alternative screening protocols were calculated. Protocol 1 represented usual practice, while Protocol 2 reflected treatment targeting using multimodal imaging. Cost-effectiveness was assessed using the net-benefit framework.

**Results:**

Protocol 1 had a total mean per patient cost of $2,013 USD and 0.148 QALYs. Protocol 2 had a total mean per patient cost of $1,519 USD and 0.153 QALYs. For a range of willingness-to-pay values, representing implicit thresholds of cost-effectiveness, the lower bound of the incremental net monetary benefit statistic was consistently greater than zero, indicating that MMCT is cost- effective compared to usual practice. The results were most sensitive to variation in the mean number of alteplase vials administered.

**Conclusion:**

In a healthcare setting where multimodal imaging technologies are available and reimbursed, their use in screening patients presenting with acute stroke to determine eligibility for alteplase treatment is cost-effective given a range of willingness-to-pay thresholds and warrants consideration as an alternative to routine practice.

## Introduction

The timely evaluation and diagnosis of ischaemic stroke is important given the narrow therapeutic time window in which intravenous recombinant tissue plasminogen activator (IV-tPA) should be administered [1]. Current protocols for evaluation and diagnosis of acute stroke recommend the use of non-contrast brain computed tomography (NCCT). However more sophisticated neuroimaging is available in the form of CT perfusion (CTP) and CT angiography (CTA). Acute CTP has been validated to identify subgroups of ischemic stroke patients who receive the most benefit from reperfusion therapy [2, 3] as well as those who may be harmed due to haemorrhagic transformation [4]. Additionally, advanced imaging can identify patients with a low probability of benefit from intravenous reperfusion therapy because their ischaemic lesion is too severe or there is minimal tissue to salvage. In this era of more personalised medicine, it is appropriate to consider the impact of targeted stroke therapy not only on individual patient outcomes but on the cost of the additional assessments to the health care system.

The cost-effectiveness of thrombolysis for acute ischaemic stroke is well established [5–7]. However, there is limited published information on the cost-effectiveness of multimodal CT (MMCT) for targeting patients suitable to receive intravenous reperfusion therapy. Young et al. [8] present a modelled economic analysis comparing MMCT to NCCT as an alternative tool for determining eligibility for endovascular therapy in a hypothetical cohort of non-haemorrhagic stroke patients using data extracted from the clinical literature. The results from their analysis showed that over a 3-month follow-up, MMCT was cost saving in comparison to NCCT (-$1,716 USD) and associated with greater quality-adjusted life-years (QALYs, 0.004). The authors concluded that given a willingness-to-pay (WTP) threshold of $100,000/QALY USD, MMCT represented a cost-effective choice. However, cost-effectiveness of MMCT has not been assessed using real-world data.

## Aims

Using data from the International Stroke Perfusion Imaging Registry (INSPIRE), administered by the University of Newcastle Stroke Research Program (UNSRP), we investigated the cost and cost-effectiveness of targeted reperfusion therapy for acute ischaemic stroke patients by comparing two alternative reperfusion treatment selection protocols. Protocol 1, representing usual practice, relies on non-contrast CT (NCCT), CT-angiography (CTA) and clinician judgment, while protocol 2 reflects treatment targeting using multimodal imaging (NCCT, CTA and CTP) to identify the ideal target for reperfusion therapy.

## Methods

**Fig 1:**
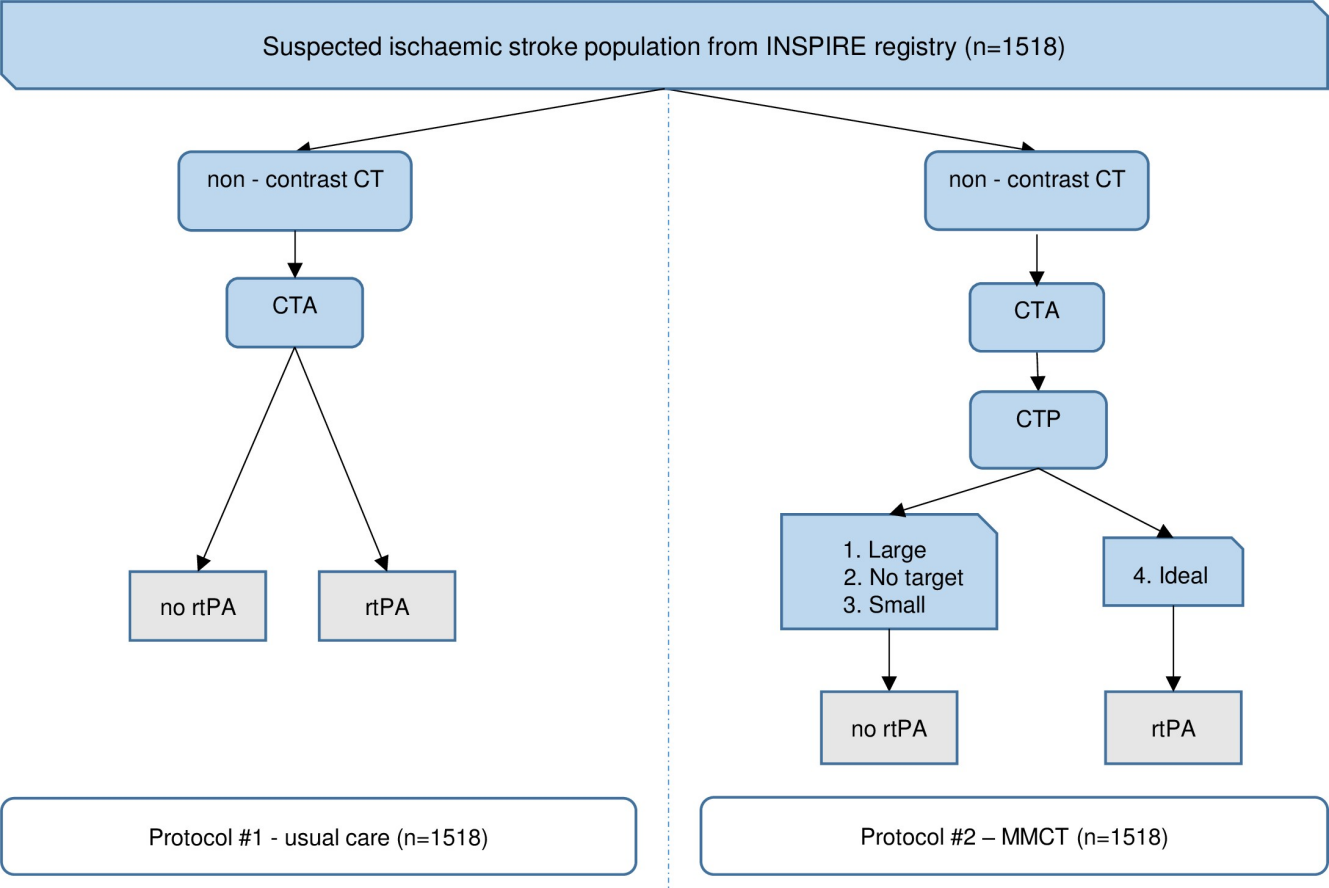
Decision analytic model structure.

We undertook a cost-utility analysis using a decision analytic framework to compare the costs and outcomes of two alternative reperfusion treatment selection protocols applied to the same population of patients with an acute ischaemic stroke presentation (Fig 1). Cost utility analysis allows comparison of interventions both within and between disease areas by using outcome measures that combine length of life and quality of life into a single summary measure, the quality adjusted life year (QALY). In this approach, states of health are assigned a health state preference or 'utility' value. The amount of time an individual spends in a given health state is multiplied by the health state preference value to calculate the quality-adjusted life-years (QALYs) gained.

The analysis was based on patient level data from INSPIRE [9]. The outcome measure was 90-day QALYs, calculated using transformed modified Rankin Score (mRS) data from INSPIRE. Resource use was estimated according to the respective treatment protocols and Australian unit costs were applied. Costs were calculated in 2016 Australian dollars and presented in 2016 US dollars using a purchasing power parity of $1 AUS = $0.69 US [10]. Cost-effectiveness was assessed using the net-benefit framework calculating an incremental net monetary benefit statistic. The time horizon for the analysis was three months post stroke event reflecting the available functional outcome assessment data included in INSPIRE. Extrapolation was not undertaken because stroke outcomes are well established at 3-months follow-up.

### Study population

Consecutive acute ischemic stroke patients presenting to hospital within 4.5 hours of symptom onset at five centres ([1] John Hunter Hospital, NSW, Australia; [2] Gosford Hospital, NSW, Australia; [3] Huashan Hospital, Shanghai, China; [4] the Second Affiliated Hospital of Zhejiang University, Hangzhou, China; and [5] Sunnybrook Health Science Centre, Toronto, Canada) between 2011–2014 were prospectively recruited to INSPIRE. Patients underwent baseline (within 4.5 hours of stroke onset) MMCT imaging and follow-up imaging at 24 hours post-stroke. Clinical stroke severity was assessed at the two imaging time points using the National Institutes of Health Stroke Scale (NIHSS). Eligible patients were treated with intravenous thrombolysis according to local guidelines and the clinical judgement of the treating physician. The modified Rankin scale (mRS) was assessed 90 days after stroke. Written informed consent was obtained from all participants, and the INSPIRE study was approved by the local ethics committees in accordance with Australian National Health & Medical Research Council (NHMRC) guidelines. The analysis was conducted in Newcastle, Australia, the site of the INSPIRE study and database. Patients undergoing endovascular procedures were not enrolled in the INSPIRE database during this time period. Retrospective CTP analysis of the patient population was conducted to classify patients into treatment or no treatment groups for the MMCT treatment targeting applied in protocol 2.

### CTP analysis and classification of patients

All perfusion imaging was post processed on commercial software MIStar (Apollo Medical Imaging Technology, Melbourne, Australia). The acute MMCT protocol is described in detail in S1 File. Acute perfusion imaging was processed using single value deconvolution with delay and dispersion correction [11]. Previously validated thresholds were applied in order to measure the volume of the acute perfusion lesion (relative delay time, DT >3 seconds) and acute infarct core (relative CBF <30%) [12]. Penumbral volume was calculated from the volume of the perfusion lesion (DT threshold >3 seconds) minus the volume of the infarct core (relative CBF threshold <30% within the DT >3sec lesion), the volume of severely hypoperfused tissue (DT >6sec) was also recorded for haemorrhage prediction. The mismatch ratio was determined as the ratio of the perfusion lesion volume (DT>3sec to the volume of the ischemic core (DT>3sec, CBF<30%).

Following post processing, patients were categorised into one of four groups [2]:

1. Large core—A large infarct core >70mL on acute CTP.
2. No target—Lack of significant perfusion lesion-core ‘mismatch’. This was determined as the measured ratio of perfusion lesion to core. If a patient had a ratio of <1.2, they were considered to have no mismatch.
3. Small lesion—A small perfusion lesion, any patient with a perfusion lesion of <5mL [13]. Overlapping patients who had a small perfusion lesion and no mismatch were considered to be in the small perfusion lesion group.
4. Ideal target—Target mismatch patients who were considered ideal candidates for reperfusion therapy. We used the DEFUSE 2 mismatch criteria of and absolute mismatch volume >15mL, mismatch ratio >1.8, a baseline ischemic core <70mL, and volume of severely hypoperfused tissue <100mL.

In the economic analysis, we assumed for protocol 1 that all patients would have one NCCT test and one CTA test. The probability of receiving rtPA reflected the proportion of treated patients observed in the INSPIRE data (Table 1). For protocol 2, we assumed all patients would receive NNCT, CTA and CTP and only patients categorised in subgroup 4 (ideal target) would receive rtPA.

**Table 1:** Patient characteristics from INSPIRE.

	1. Large	2. No target	3. Small	4. Ideal
N (Total 1518)	228	504	408	378
Proportion died	37%	18%	2%	8%
Treated N (%)	161 (70%)	354 (70%)	166 (40%)	256 (70%)
Mean age				
*Treated*	56	63	52	73
*Not treated*	59	59	66	72
% Males				
*Treated*	49%	54%	58%	46%
*Not treated*	58%	61%	51%	54%
Mean transformed utility				
*Treated*	0.26	0.60	0.77	0.61
*Not treated*	0.25	0.63	0.83	0.52
*Difference (95% UI)*				(*0*.*0899*, *0*.*0940*)

### Measurement of outcomes

From INSPIRE, we extracted 90-day modified Rankin Scale (mRS) data for each patient in the dataset. These health outcomes, including mortality, were transformed into utilities using the algorithm published by Ramos-Goni et al [14]. In the absence of Australian specific values in the algorithm, we used the value set for the UK population to calculate mean utilities [15]. We calculated individual utility values for each patient in the dataset. An mRS score of 6, indicating death was appropriately mapped to a utility of zero. Mean utility was calculated for the whole cohort, for each of the four treatment target groups (1. Large core, 2. No target, 3. Small lesion, 4. Ideal target) and for the treated and untreated subgroups within the four groups (Table 1).

For protocol 1, we used the patient level utility values calculated using the method described above. These values reflect the expected utility for a cohort of patients whose actual treatment determination and outcome align to protocol 1. The outcomes associated with protocol 2 in the decision analysis, were directly aligned with treatment status. Due to variation in local physician preference and institutional guidelines, some patients in group 4 were not treated and some patients in categories 1, 2 and 3 were treated [2]. Therefore, we undertook the following steps for calculating mean utility for the cohort described under protocol 2:

- Treated patients in group 4 retained their transformed utilities (calculated as for protocol 1). Untreated patients in group 4 had their utility values imputed using the mean utility calculated for the treated subgroup (calculated under protocol 1).
- Untreated patients in groups 1, 2 and 3 retained their transformed utilities (calculated as for protocol 1). Treated patients in groups 1, 2 and 3 had their utility values imputed from the mean utility values calculated for the untreated subgroups from each group (1, 2 and 3,) respectively.

The average number of QALYs for each of the protocols was calculated by multiplying the mean utility across the cohort by 90/365.

### Measurement and valuation of costs

For each patient in the INSPIRE cohort and for each protocol, we calculated the cost of imaging and treatment, valued using the 2016 Medicare Benefit Schedule fee and the cost of treatment with alteplase. Treatment cost was calculated as the cost of two packs (1 x 50 mg vial, 1 x 50 mL inert diluent), reflecting the recommended dose of 0.9 mg/kg body weight (maximum of 90 mg) and including an allowance for wastage. In protocol 1, the costs included NCCT imaging for all patients and the cost of treatment with 50mg alteplase if indicated in the dataset. In protocol 2, the costs included MMCT imaging for all patients and the cost of treatment with 50mg alteplase only for category 4 (Ideal) patients.

### Statistical analysis

We undertook the cost-effectiveness analysis from the health care funder perspective. All analyses were carried out using Microsoft Excel 2013. Mean costs and outcomes were compared across each of the two alternative protocols. We used the net-benefit framework to report the cost-effectiveness results, calculating an incremental net monetary benefit (iNMB) statistic. The iNMB in this analysis was calculated as the difference in mean QALYs per patient multiplied by the maximum willingness-to-pay for a QALY, minus the difference in mean cost per patient. We used the cost-effectiveness threshold range $20,690 –$62,069 USD as the lower and upper limits of the maximum willingness to pay for a QALY (W), reflecting implicit threshold ranges reported in the literature [16].

Cost-effectiveness results presented using the net- benefit framework have the advantage of being simple to interpret. If the lower bound of the incremental net benefit statistic is greater than zero (iNMB>0), the intervention in question should be adopted [17].

### Uncertainty and sensitivity analysis

We accounted for uncertainty in the analysis using nonparametric bootstrapping to derive uncertainty intervals (UI) around the point estimates. We also conducted sensitivity analysis to examine the impact of variation in the treatment cost and tested the impact of using difference preference weights to generate utility values.

## Results

### Study population characteristics

The INSPIRE data set included age, sex, resource use frequency data and outcome variables for a total of 1,518 stroke patients. Patient characteristics and calculated utilities according to MMCT assigned category, representing protocol 1, are presented in Table 1. The utility values of the treated and non-treated sub groups do not vary significantly across the categories with the exception of category 4 (ideal) where the calculated utility value for treated patients is higher (mean utility difference *p* < .05).

### Costs and cost-effectiveness

**Fig 2:**
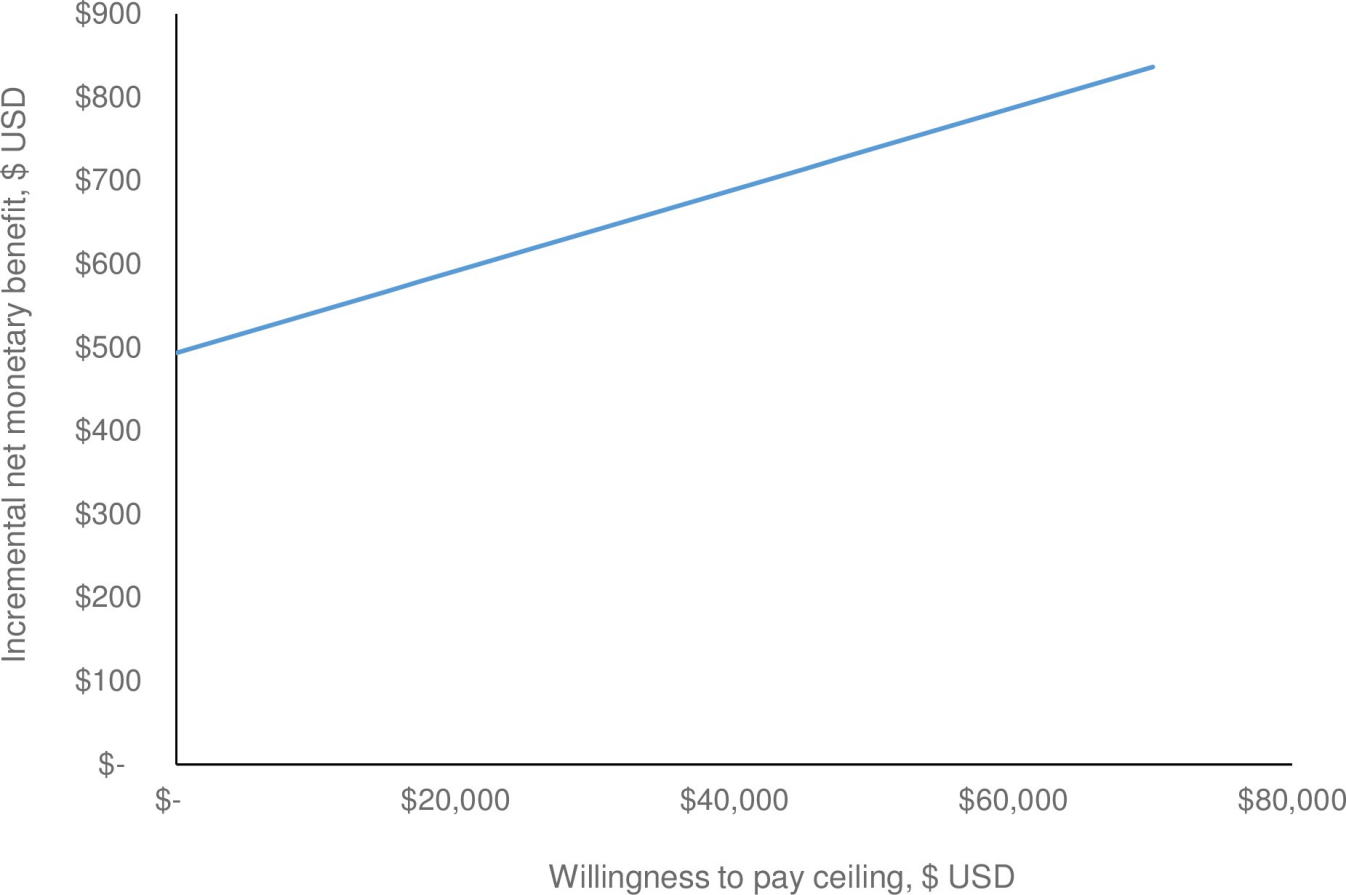
Net benefit curve.

Mean total costs per patient were $2,013 USD (95% UI *$1*,*951 to $2*,*061*) for protocol 1 and $1,519 for protocol 2 (Table 2). The difference in cost is driven by a reduction in the number of patients receiving treatment under protocol 2. Mean total QALYs per patient were calculated to be 0.148 *(95% UI 0*.*144*, *0*.*152)* for protocol 1 and 0.153 *(95% UI 0*.*152*, *0*.*155)* for protocol 2. The mean incremental net monetary benefit (iNMB) between the two protocols, at willingness-to-pay thresholds of $20,690 USD per QALY and $62,069 USD, were $640 (95% UI *$479*, *$705*) and $935 (95% UI *$526*, *$1*,*076)*, respectively (Table 2). Since the lower bound of the iNMB, for either WTP threshold, is greater than zero, these results show that MMCT is the cost-effective alternative. The net benefit curve (Fig 2) depicts the expected change in net benefit associated with increasing WTP thresholds.

**Table 2:** Mean costs per patient, utility values and QALYs.

	Protocol 1 –Current treatment selection		Protocol 2 –MMCT treatment targeting	
	Mean cost (USD)		Mean cost (USD)	
Imaging	$486		$904	
Treatment	$1,527	*($1*,*465*, *$1*,*582)*	$616	
Total cost (*95% UI*)	$2,013	*($1*,*951*, *$2*,*061)*	$1,519	
90 day QALYs (*95% UI*)	0.148	*(0*.*144*, *0*.*152)*	0.153	(*0*.*152*, *0*.*155*)
Incremental net monetary benefit (iNMB)				
WTP threshold $20,690 USD *(95% UI)*	$640	*($479*, *$705)*		
WTP threshold $62,069 USD *(95% UI)*	$935	*($526*, *$1*,*076)*		

Abbreviations: QALY, quality adjusted life year; UI, uncertainty interval; WTP, willingness to pay; USD, US dollars

### Sensitivity analysis

**Fig 3:**
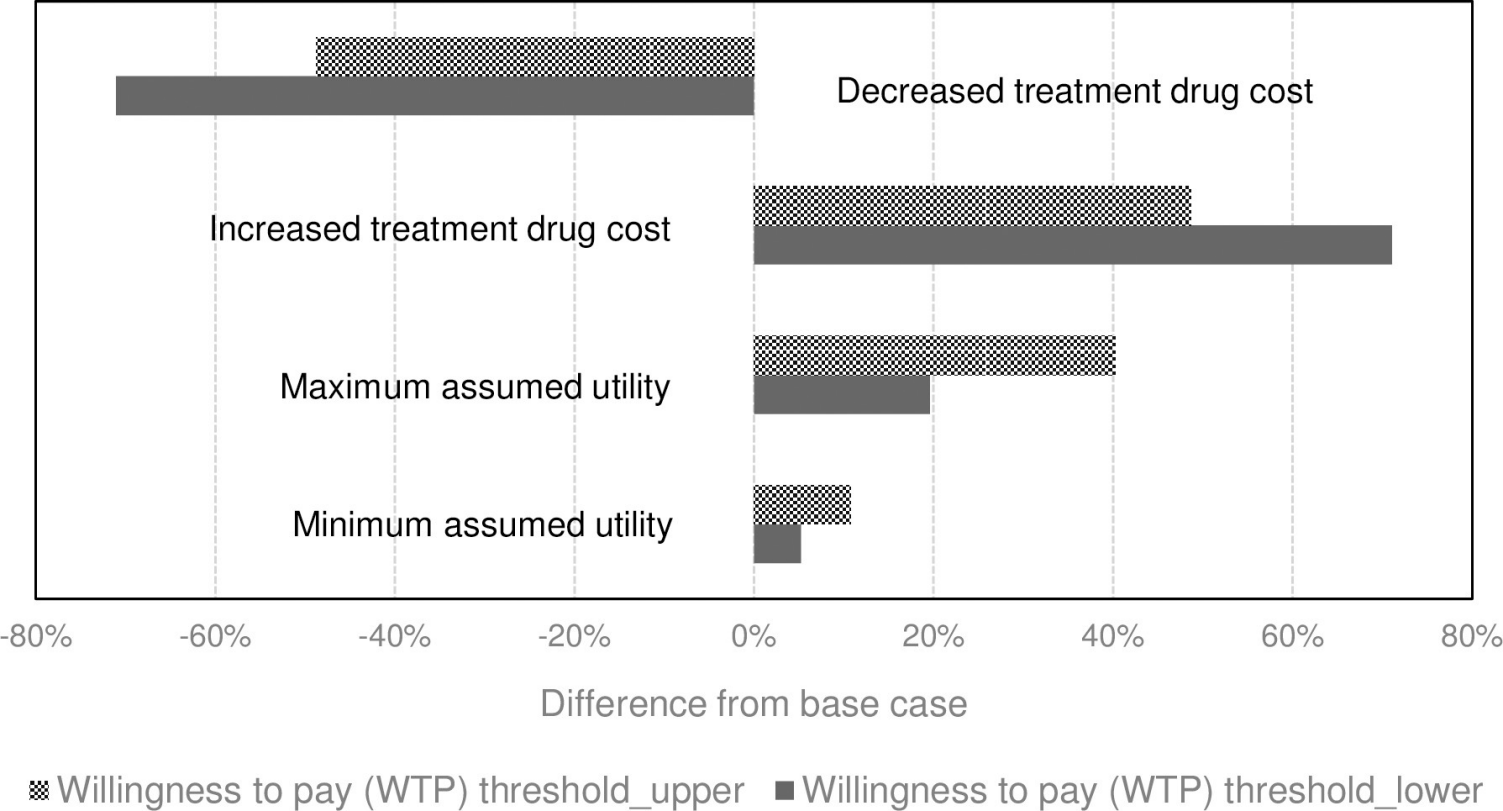
Sensitivity analysis results.

The results were most sensitive to variation in the treatment cost, modelled by varying the mean number of vials of alteplase administered. The higher the treatment cost, the greater the incremental net benefit (Fig 3). Varying the utilities between the minimum and maximum range, reflecting different country preference weights, also resulted in higher iNMB estimates (Fig 3).

## Discussion

Our economic analysis based on data from INSPIRE, showed that the additional costs to the health system incurred by MMCT are offset due to fewer patients requiring treatment with IV-rtPA. That is, screening with MMCT is cost saving. Furthermore, the analysis showed that these cost savings are achieved with no loss in utility. This resulted in a positive incremental net monetary benefit when compared against a range of maximum willingness-to-pay thresholds for a QALY [16]. Our analysis results are consistent with the findings from the only other published economic analysis evaluating MMCT identified in the literature (5). The Young et al. analysis showed that MMCT had lower costs, greater QALYs and was the cost-effective choice 100% of the time for a willingness-to-pay of $100,000/QALY USD. The main strength of our analysis is the use of real world, patient level data captured across multiple international sites contributing to INSPIRE. The data registry provided information on stroke severity and respective treatment groups, as well as mRS values from which we could calculate utility scores.

In the present study we divided patients into one of four groupings based on previous work suggesting futile treatment with alteplase in large ischemic cores, a good natural history of patients with small or no perfusion lesion (which includes possible stroke mimics), patients not suspected to benefit from alteplase (no mismatch), and 'ideal' alteplase candidates (target mismatch). The clinical outcome information from the QALY reinforce these groupings and suggest that considering alternate forms of reperfusion therapy for patients with large ischemic cores or no mismatch, such as endovascular therapy, may be ideal. Alteplase therapy poses several risks, particularly of haemorrhagic transformation, which is most amplified in select groups of patients, those with large infarct cores. However, this notion is currently being challenged in patients receiving endovascular therapy. In this study, patients with an ideal imaging target demonstrated the greatest cost effective treatment response to intravenous thrombolysis due to a dramatic reduction in their disability at 3 months. Our data suggests that a targeted treatment approach where patients with an ideal imaging profile with alteplase and/or endovascular therapy, if appropriate, leads to improved clinical outcomes over an approach of treating all suspected ischemic stroke patients [2, 18]. Such a targeted treatment approach may result in the best possible patient outcomes and be particularly cost effective, due in large part to the role of imaging selection in patient management and the improved patient outcomes with or without interventions, resulting in long term cost benefits through reduced burden of care.

A number of limitations should also be recognised. Not every country, health district or hospital will have access to the imaging technologies employed in the analysis and the cost profile reflects the study jurisdiction. Hence, we recognise that these results will not have universal applicability and that targeting treatment using MMCT may not be cost saving in other settings. Further, there are a number of technical limitations. First, the omission of the cost of hospital stay. Any variation in length of stay or intensity of resource use was therefore not captured and may bias the findings. Second, we derived the utility values based on a transformation of the mRS data. Ideally, utility values should be directly elicited. Third, patients in this analysis were divided into groups based on imaging profiles and the results do not stem from patients’ random allocation to treatment. The probability of receiving treatment under Protocol 1 will have included such factors as patient age and co-morbidities. The patient characteristics data that were available (Table 1) show that the mean age of the treated and untreated cohorts in each of the groups do not systematically bias the results. While data on patient co-morbidities were unavailable for this analysis, the use of propensity score matching based on these data would be an option to explore in any future analysis to mitigate the risk of bias.

## Conclusion

Compared to current practice, defined as non-contrast CT (NCCT), CT-angiography (CTA) and clinician judgment, MMCT is a cost effective screening tool when used to target patients for rtPA treatment. This analysis showed that under conservative scenarios, MMCT can be cost saving with no loss in clinical or quality of life outcomes.

## Supporting information

S1 File Acute multimodal CT protocol. (PDF)
