# Supplementary material for: Cost-effectiveness of targeted thrombolytic therapy for stroke patients using multi-modal CT compared to usual practice
Source: PLoS One. 2018 Oct 23;13(10):e0206203. doi: 10.1371/journal.pone.0206203 (PMC6198974; doi:10.1371/journal.pone.0206203)
Supplement: S1 File — (PDF) [file pone.0206203.s001.pdf]

S1: Technical Appendix

Acute Multimodal CT Protocol

Participants recruited at John Hunter Hospital were scanned using a Toshiba Aquilion 320-slice CT scanner (Toshiba Medical Systems; Tokyo, Japan). A total of 19 acquisitions occurred in 60 seconds. Forty mL of contrast agent (Ultravist 370; Bayer HealthCare; Berlin, Germany) was injected at 6 mL/s, followed by 30 mL of saline. Participants recruited at Gosford Hospital were scanned using a 64 detector GE lightspeed (GE Healthcare, Waukesha, Wisconsin). A total of 19 acquisitions occurred in 54 seconds. Forty five mL of contrast agent (Ultravist 370; Bayer HealthCare; Berlin, Germany) was injected at 6 mL/s. Participants recruited at Huashan Hospital were scanned using a Brilliance iCT 126-slice CT scanner (Philips Medical Systems, Cleveland, Ohio). A total of 23 acquisitions occurred in 60 s. Forty mL of the same contrast was injected at 5 mL/s, followed by 20 mL saline. Participants recruited at the Second Affiliated Hospital of Zhejiang University were scanned using a Definition Flash duel source CT scanner (Siemens, Erlangen, Germany) with 64 detectors. A total of 10 acquisitions occurred in 60 seconds. Fifteen mL of contrast was injected at 4mL/s followed by 20mL of saline. Participants recruited at Sunnybrook Health Sciences Center underwent an acute CT with a 64-row CT scanner (GE Healthcare, Waukesha, Wis). Baseline and 24-hour CT angiogram parameters were as follows: 0.7 mL/kg iodinated contrast agent up to a maximum 90 mL (Omnipaque 300 mg iodine/mL; GE Healthcare, Piscataway, NJ); 5-10-second delay; 120 kVp; 270 mA; rotation, 1 second; 1.25-mm- thick sections; table speed, 20.62 mm per rotation. Biphasic CT perfusion protocol from basal ganglia to the lateral ventricles: 80 kVp; 150 mA: collimation, 8 x 5 mm; rotation, 1 second for 45 seconds followed by 6 further acquisitions 15 seconds apart for a total of 135 seconds. Iodinated contrast agent 0.5 mL/kg (maximum 50 mL) at 4 mL/sec was administered 5 seconds prior to start of the sequence. All acute perfusion imaging acquired 60mm or more of coverage.
